# 

**Published:** 1891-04

**Authors:** 


### CONTENTS.

### COMMUNICATIONS.

Report of Case of Cancrum Oris .... 161
A New Antiseptic in Dentistry .... 163
Atomic Weights .... 165
Brain and Fingers .... 167

### SELECTIONS.

Dr. Holmes on Specialism .... 168
The Ethics of Experimentation upon Living Animals .... 170
Patent Rights .... 171
The Weariness of Dental Practice .... 173
The Blood Supply of Nerves .... 175
Spontaneous Generation .... 176
Disinfection of the Hands .... 177
The Progress of Surgery in 1890 .... 178
Management of Primary Dentition .... 181
Prophylaxis .... 182
Dr. Henry J. Bigelow, and the Discovery of Anæsthesia .... 183
Solution for the Eczema of Dentition .... 184
An Animated Manikin .... 185
The Causes of Dyspepsia .... 185
Items .... 186
Deciduo—Permanent Teeth .... 186
Italian Quacks and Lymph .... 187
The Influence of Organic Acids upon the Salivary Conversion of Starch .... 191
Coffee .... 193
Concentrated Foods .... 194
Sir Richard Quain .... 194
Cocaine Again .... 195
Bacillary Partnerships .... 197

### SOCIETIES.

Illinois State Meeting .... 199
Northern Ohio Dental Association .... 200
American Medical Association .... 201
Idontograph Society, Pittsburgh, Pa .... 203

### EDITORIAL.

Fifty Ologies that concern the Dental Profession .... 203
What is the Explanation? .... 205
How to Clean a Mixing Slab .... 207
Recurring Luxation of the Lower Jaw .... 208

### OBITUARY.

The Death of Dr. C. R. Coffin .... 212

